# Kinematic Investigations of a Novel Flapping Actuation Design with Mutually Perpendicular 3 Cylindrical Joint Approach for FW-Drones

**DOI:** 10.3390/biomimetics8020160

**Published:** 2023-04-17

**Authors:** Spoorthi Singh, Mohammad Zuber, Mohd Nizar Hamidon, Adi Azriff Basri, Norkhairunnisa Mazlan, Kamarul Arifin Ahmad

**Affiliations:** 1 Department of Aerospace Engineering, Faculty of Engineering, Universiti Putra Malaysia (UPM), Serdang 43400, Selangor, Malaysia; 2 Department of Mechatronics Engineering, Manipal Institute of Technology, Manipal Academy of Higher Education (MAHE), Manipal 576104, Karnataka, India; 3 Department of Aeronautical & Automobile Engineering, Manipal Institute of Technology, Manipal Academy of Higher Education (MAHE), Manipal 576104, Karnataka, India; 4 Institute of Nanoscience and Nanotechnology, Universiti Putra Malaysia (UPM), Serdang 43400, Selangor, Malaysia; 5 Institute of Advanced Technology (ITMA), Universiti Putra Malaysia (UPM), Serdang 43400, Selangor, Malaysia; 6 Aerospace Malaysia Research Centre (AMRC), Universiti Putra Malaysia (UPM), Serdang 43400, Selangor, Malaysia

**Keywords:** flapping wing drones, rotary to flapping conversion, cylindrical joint dynamics, kinematic investigations of FWMAV

## Abstract

The transmission mechanism of artificial flapping-wing drones generally needs low weight and the fewest interconnecting components, making their development challenging. The four-bar Linkage mechanism for flapping actuation has generally been used till now with complex and heavy connecting designs, but our proposed novel perpendicularly organized 3-cylindrical joint mechanism is designed to be unique and lighter weight with smooth functioning performance. The proposed prototype transforms the rotary motion of the motor into a specific angle of flapping movement, where the dimensions and specifications of the design components are proportional to the obtained flapping angle. Power consumption and flapping actuation can be monitored by adjusting the motor’s rotational speed to control the individual wing in this mechanism. The proposed mechanism consists of a crank with three slightly slidable cylindrical joints perpendicularly arranged to each other with a specified distance in a well-organized pattern to produce a flapping movement at the other end. In order to examine the kinematic attributes, a mathematical process approach is formulated, and kinematic simulations are performed using SIMSCAPE multibody MATLAB, PYTHON programming and COMPMECH GIM software. The proposed invention’s real-time test bench prototype model is designed, tested and analyzed for flapping validation.

## 1. Introduction

Investigators have made significant progress in understanding the extraordinary flying uniformity and drivability of flapping-winged creatures. Insect flight is dependent on the alignment of their flapping wings with the coordination of their movements. Because of the remarkable flight technique used by insects/birds, scientists have found encouragement in bio-inspired flying mechanisms to develop feasible as well as productive human-made flyable structures. Especially in comparison to rotary and fixed type mechanisms, the flapping wing process is the most durable, possibly since its potential-energy is transferred through a larger-chord, pulsating from a limit of zero thrust that enhances both ends of the flapping angular position to a high level of sustainment. One of the most notable characteristics of the mentioned flapping frameworks is that they are dynamic oscillating structures with sufficient flexibility. Substantial progress has been achieved by various researchers in grasping the incredible flying consistency and maneuverability of hummingbirds and flapping-winged insects/flies. Usually, movements of insects rely on the co-synchronization of their flapping wings during its flight. Because of the impressive flight technique involved in insects and birds, various scientists have discovered inspiration in biological flying systems (for example: microbat [1], Lung-Jieh Yang’s MAV [2], Bristol’s PCR prototype [3], Lung-Jieh Yang’s Golden Snitch [4,5,6]) to find workable and efficient man-made flying mechanisms. A flappable wing operation is more durable as contrasted to the rotational or static types, possibly as its motion is distributed across a longer cord, fluctuating from a zero thrust restriction which boosts either ends of said fluttering inclination to a significant importance. The amazing feature of the fluttering frameworks is because they are meant to be a versatile, effective, and stretchy oscillation mechanism.

The Thunder I model merges its varied configurations well with its concept of the cranked mechanism, excluding the usage of guidance bar instead of sliding system. To achieve a uniform fluttering movement along both sides, a 6-bar structure comprising 14 hinged elements is employed, which results in more lift. The KUBEETLE-S model employs a 4-bar connection with a pulley–string structure to provide clap and flinging effects during left/right stroking modifications, resulting in increased lift. The bigger stroke length for the flapping-wing as well as the increased masses lead to a larger lift with the longest flying endurance possible. A little imitating bird/creature has a Cranks connected rocker arrangement which is composed of short, flexible parts. As a result of its flaps, the primary/secondary struts of the wings are displaced, modifying the camber position or width with mid-chord speeds and thereby changing the lift and thrust rates and the better version of flapping actuation mechanism is obtained [7,8,9,10,11,12]. The adherence of the principal component modifies the apparent wingspan across to the fluttering plane to ensure stableness under rise in harmonic fluctuations. We note the rise in average performance brought on by better wing responsiveness. The Saturn concept uses a revolving crank-shaft with string-based dual mechanical connection with two strings which are controlling the swings of the respective pulley system at the wing hinge. The wing swing angle distribution is periodical; however, comparing to the linkage-based system, the amplitude of the tip velocity is greatly reduced. Because of the construction process and wing pitch maneuverability, such as rotation and twist distribution, it is feasible to manage the pitch, roll, and yaw axes. The design of the rotorcraft’s swashplate assists to deliver sensor readings to actuators with a significantly decreased slightly than the flapping frequency. Throughout wing transmembrane camber inversion, the impact of flapping stroke generates the greatest outcomes due to the various wing modelling approaches. The importance of fluttering actuations and wing orientation to the generation of lift is evident from the above-described studies and recent literature review of prototype design mechanisms. Due to the complexity of designing and fabricating lighter, more efficient, and smaller FWMAVs, an efficient design approach is essential to obtain a superior conclusion [13,14,15].

There are several well-known flying vehicles that use various flapping actuation mechanisms to make them fly. Nevertheless, many of them make use of sliding connections made of rods, rockers or couplers for the system that is powered by an electric motor. There is currently no simple flapping actuation mechanism with triple cylindrical joints axes arranged in a perpendicular direction to each other that can be used for flapping generation. It is critical to reduce the complexity of the flapping actuation mechanism designs, which in turn reduces the overall weight of the system as a consequence. We developed a novel and less complex mechanism for flapping actuation called the “Crank driven-triple cylindrical slidable and rotatable joint” mechanism arrangement. The fundamental connection for converting rotary motion to specific angular movement of flaps in this case is a horizontal path formed by three cylindrical joints, where the axes of all three joints are perpendicular to each other. Using joint coordinates and homogeneous transformation matrices, the geometry of the system is described. A crank-driven triple cylindrical connection arrangement is unique and easily designable with minimal components.

## 2. A Description of the Kinematic Analysis Process

**Figure 1:**
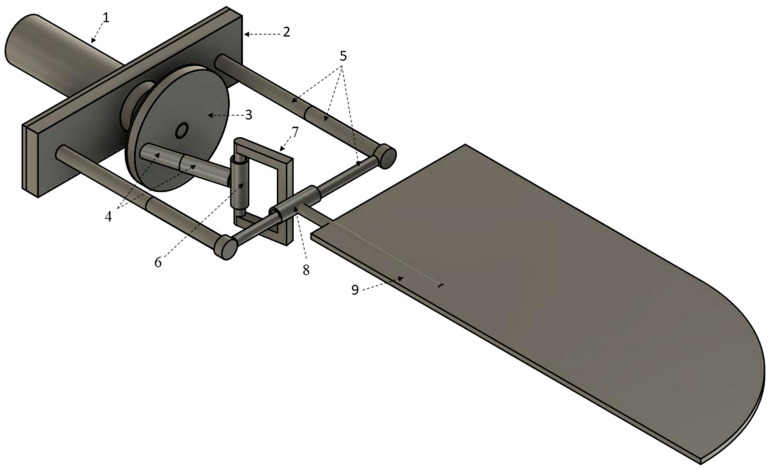
Novel Flapping Actuation Mechanism Design.

In this mechanism, generated motion and flapping is considered for the all-cylindrical joints C_n_ of the proposed mechanism design. The mechanism is arranged in freely rotatable and slightly slidable joint, where in the first cylindrical joint one end of the outer cylinder is connected to a crank and another end is open. Similarly, the one end of the indirectly connected link inside the cylindrical is open/freely movable and another end of it is connected to second cylindrical joint as shown in Figure 1. The rotational force reaction acting on the connected links are elaborated in this paper furthermore. Using the Denavit–Hartenberg nomenclature, the morphology of our proposed design joints is described. This nomenclature is based on 4 × 4 homogeneous transformation matrices and joint-coordinates (defining the relative position of links) in accordance with the approach described in the paper [13]. Moreover, this section describes the geometry of the mechanism links. Since it can be seen easily that the structure of the obtained connections is analogical to the structure of the linkages, which are modelled in the form of open-loop kinematic joint coordinates, the procedure regarding formulating equations of the connections with motion can be analogical to the approach which is used in the case of the kinematic and dynamic analysis of the flapping actuation mechanism.

### Model Description and Working Principle

**Figure 2:**
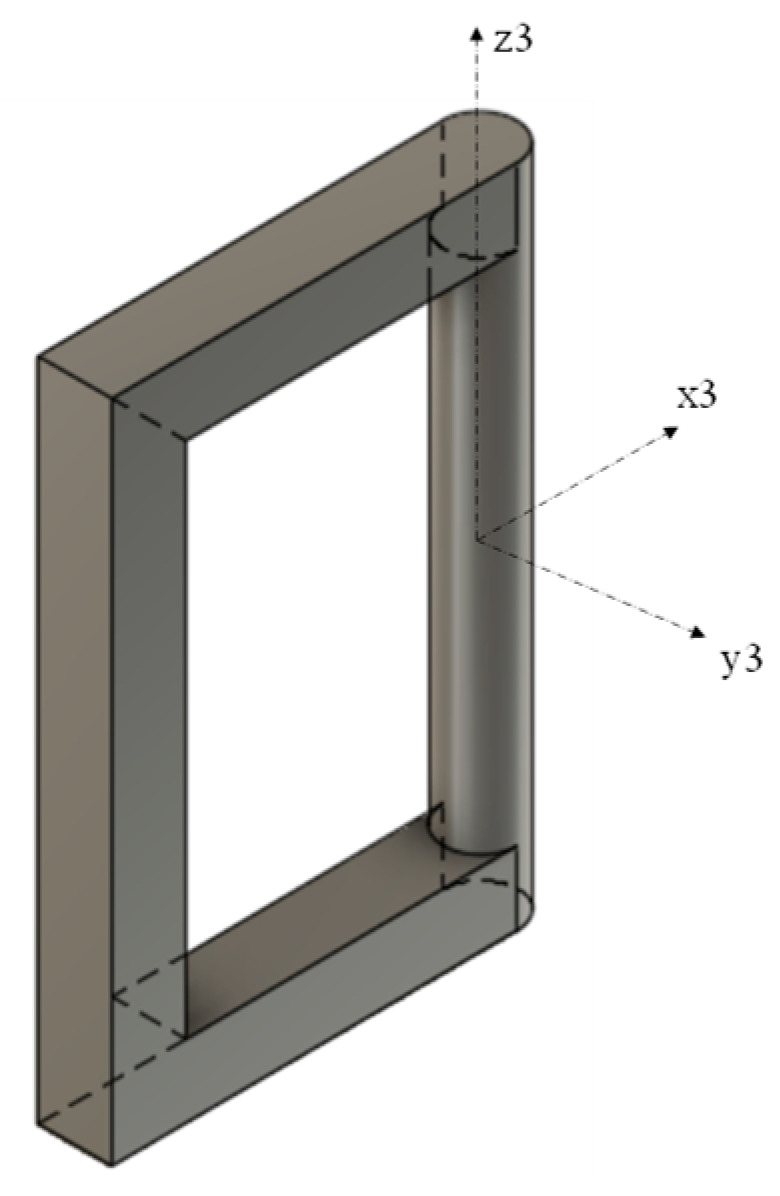
Rectangular frame structure.

**Figure 3:**
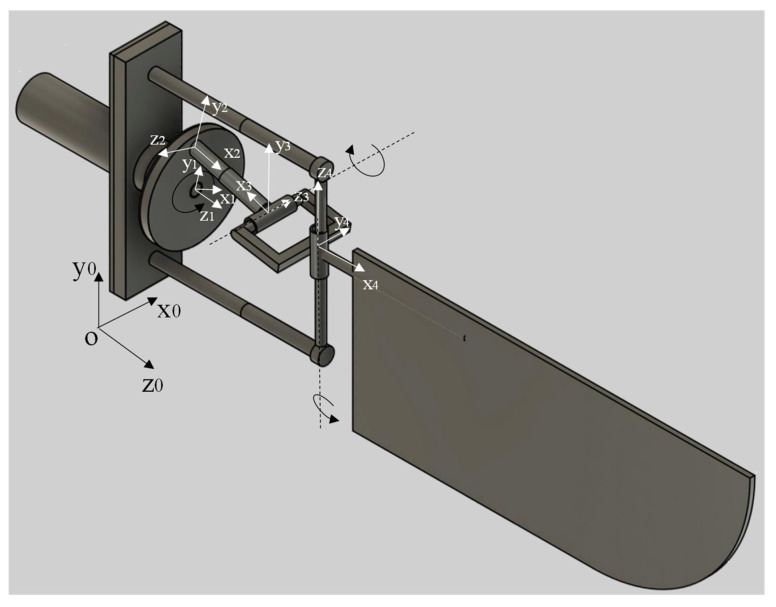
Coordinate systems that are presumed.

**Figure 4:**
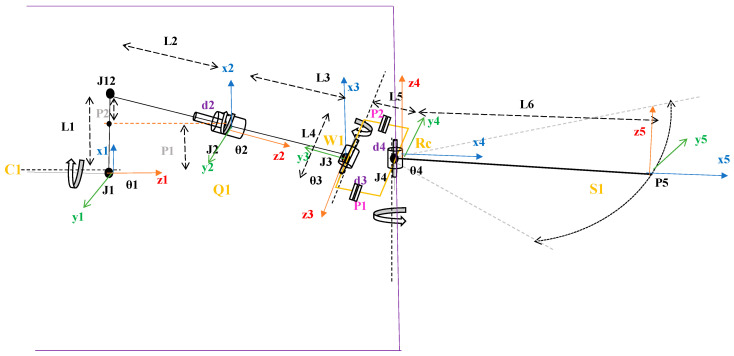
Kinematic structure of the flapping actuation mechanism.

The kinematic model (for one wing) of the proposed mechanism is shown in Figure 1. It is composed of: 1—Motor, 2—Base frame, 3—Crank, 4—Cylindrical joint c1, 5—Supporting frame, 6—Cylindrical joint c2, 7—Rectangular frame, 8—Cylindrical joint c3, 9—wing. The rectangular frame is highlighted in Figure 2 below. The following symbols were used in the kinematic diagram to describe the mechanism motion sequence as shown in Figure 3 and Figure 4. X, Y, Z are the coordinate axes with respect to the joint J, where L indicates the length of the connected link, d indicates the linear distance travelled, C is the crank, S is the joint between the crank and rod, Rc is the rectangular frame, W is the wing link joint at J4, and Wt is the wing tip.

Operation of the mechanism used in the model occurs as follows: the battery-powered electric motor as shown in Figure 1 drives the crank wheel of the mechanism. The link L2 is a portion of outer cylinder connected to crank with an angle of θx (i.e., the angle if connected link at joint 2), where the other end is linked to inner link through a cylindrical joint assumed to be c1. The continuity of the inner link is attached directly to the outer face of cylindrical joint c2 in a perpendicular way. A rectangular frame with a cylindrical shape in one of its links and the remaining 3 links are of square shape, is shown in Figure 2. The cylindrical shape of the rectangular frame structure is considered to be inner link of the cylindrical joint c2 linked to outer link of c2. The opposite side square shape of the rectangular frame structure is attached in perpendicular to the outer face of cylindrical joint c3. The inner link of the joint c3 is a supporting frame which is fixed as shown in Figure 1. The last link L5 is a wing connecting link, attached to the outer face of cylindrical joint c3.

## 3. Kinematics of the Mechanism

**A.**  **Forward kinematics**

The crank-driven mechanism’s motion is characterized by the joint coordinate ψ (Jn), which is an aspect of the believed common coordinate vector [16,17] used in Figure 3, where J represents joint coordinates of the system and n represents its position.

Assumed coordinates at joints C1, Q1, W1, Rc, and S1 are as shown below and also in Figure 4: (note: The following derivations are performed by considering the stated nomenclature in this paper.)

$$q^{\left(J1\right)}={\left\{q_{C1}{}^{\left(J1\right)}\right\}}_{C1=1}=\psi^{\left(J1\right)}$$

$$q^{\left(J2\right)}={\left\{q_{Q1}{}^{\left(J2\right)}\right\}}_{Q1=1}=\psi^{\left(J2\right)}$$

However, P1 ׀׀ P2, considered to be single link L5, as the rectangle component at its one end performs as a supporting inner link for cylindrical joint J3 to rotate along z3 axes. The another end the rectangle component is connected perpendicular to outer structure of cylindrical joint J4. Hence. the whole rectangular component is here considered to be $R_{c}$. The tip of the wing is considered to be P5.

$$q^{\left(J3\right)}={\left\{q_{W1}{}^{\left(J3\right)}\right\}}_{W1=1}=\psi^{\left(J3\right)}$$

$$q^{\left(J4\right)}={\left\{q_{R_{c}}{}^{\left(J4\right)}\right\}}_{R_{c}=1}=\psi^{\left(J4\right)}$$

$$q^{\left(P5\right)}={\left\{q_{S1}{}^{\left(P5\right)}\right\}}_{S1=1}=\psi^{\left(P5\right)}$$

The DH Table obtained for the above shown mechanism, (i.e., Figure 4) is as shown in Table 1 below. (note: multiplication sign is indicated as star sign “∗“, below)

We tried to obtain the homogeneous transformation matrices from the DH Table and assumed the coordinate nodes linked to the standard reference were computed as follows: (note: “cos” and “sin” are indicated by “c” and “s” below)

$$T_{1}^{0}=\left[\begin{array}{ccc} c\theta 1 & -s\theta 1.c(0) & s\theta 1.s(0)\\ s\theta 1 & c\theta 1.c(0) & -c\theta 1.s\left(0\right)\\ \begin{array}{c} 0\\ 0 \end{array} & \begin{array}{c} s(0)\\ 0 \end{array} & \begin{array}{c} c(0)\\ 0 \end{array} \end{array}\begin{array}{c} \left(P1\right).c\theta 1\\ \left(P1\right).s\theta 1\\ \begin{array}{c} P2*\tan(\varphi 1)\\ 1 \end{array} \end{array}\right]$$

$$T_{2}^{1}=\left[\begin{array}{ccc} c\theta 2 & -s\theta 2.c(-90˚) & s\theta 2.s(-90˚)\\ s\theta 2 & c\theta 2.c(-90˚) & -c\theta 2.s\left(-90˚\right)\\ \begin{array}{c} 0\\ 0 \end{array} & \begin{array}{c} s(-90˚)\\ 0 \end{array} & \begin{array}{c} c(-90˚)\\ 0 \end{array} \end{array}\begin{array}{c} \left(-P1\right).c\theta 2\\ \left(-P1\right).s\theta 2\\ \begin{array}{c} L3+d3\\ 1 \end{array} \end{array}\right]$$

$$T_{3}^{2}=\left[\begin{array}{ccc} c(-90˚+\theta 3) & -s(-90˚+\theta 3).c(-90˚) & s(-90˚+\theta 3).s(-90˚)\\ s(-90˚+\theta 3) & c(-90˚+\theta 3).c(-90˚) & -c(-90˚+\theta 3).s\left(-90˚\right)\\ \begin{array}{c} 0\\ 0 \end{array} & \begin{array}{c} s(-90˚)\\ 0 \end{array} & \begin{array}{c} c(-90˚)\\ 0 \end{array} \end{array}\begin{array}{c} \left(L5\right).c(-90˚+\theta 3)\\ \left(L5\right).s(-90˚+\theta 3)\\ \begin{array}{c} \pm d3\\ 1 \end{array} \end{array}\right]$$

$$T_{4}^{3}=\left[\begin{array}{ccc} c\theta 4 & -s\theta 4.c(0) & s\theta 4.s(0)\\ s\theta 4 & c\theta 4.c(0) & -c\theta 4.s\left(0\right)\\ \begin{array}{c} 0\\ 0 \end{array} & \begin{array}{c} s(0)\\ 0 \end{array} & \begin{array}{c} c(0)\\ 0 \end{array} \end{array}\begin{array}{c} L6.c\theta 4\\ L6.s\theta 4\\ \begin{array}{c} \pm d4\\ 1 \end{array} \end{array}\right]$$

where $c\theta n=Cos\psi^{\left(Jn\right)}$ and $s\theta n=Sin\psi^{\left(Jn\right)}$

Hence, the position of the flapping link is related to its initial reference position (P*n*) by

$$T=T_{1}^{0}*\;T_{2}^{1}*T_{3}^{2}*T_{4}^{3}$$

The flapping lever position and posture of during its actuation are characterized by the generated matrix in the following way, in accordance with the homogeneous transformation obtained from the DH table.

$$T=\left[\begin{array}{cccc} R11 & R12 & R13 & PX\\ R21 & R22 & R23 & PY\\ R31 & R32 & R33 & PZ\\ 0 & 0 & 0 & 1 \end{array}\right]$$

where *T* is a 4 × 4 transformation matrix whose elements are given by

R11=[c(θ1+θ2)∗sθ3∗cθ4]+[s(θ1+θ2)∗sθ4]

R12=[−c(θ1+θ2)∗sθ3∗sθ4]+[s(θ1+θ2)∗cθ4]

R13=[c(θ1+θ2)∗cθ3]

$$\begin{array}{c} \mathrm{PX}=[c\left(\theta 1+\theta 2\right)*s\theta 3*L6*c\theta 4]+[s\left(\theta 1+\theta 2\right)*L6*s\theta 4]+[d4*c\left(\theta 1+\theta 2\right)*c\theta 3]+\\ \left\{\left[L5*c\left(\theta 1+\theta 2\right)*s\theta 3\right]-\left[d3*S\left(\theta 1+\theta 2\right)\right]+P1\left[c\theta 1-c\left(\theta 1+\theta 2\right)\right]\right\} \end{array}$$

R21=[s(θ1+θ2)∗sθ3∗cθ4]+[c(θ1+θ2)∗sθ4]

R22=[−s(θ1+θ2)∗sθ3∗sθ4]+[c(θ1+θ2)∗cθ4]

R23=[s(θ1+θ2)∗sθ3]

$$\begin{array}{c} \mathrm{PY}=[s\left(\theta 1+\theta 2\right)*s\theta 3*L6*c\theta 4]+[c\left(\theta 1+\theta 2\right)*L6*s\theta 4]+[d4*s\left(\theta 1+\theta 2\right)*s\theta 3]+\\ \left\{\left[L5*s\left(\theta 1+\theta 2\right)*s\theta 3\right]+\left[d3*c\left(\theta 1+\theta 2\right)\right]+P1\left[s\theta 1-s\left(\theta 1-\theta 2\right)\right]\right\} \end{array}$$

R31=[cθ4∗cθ3]

R32=[−cθ3∗sθ4]

R33=−sθ3

PZ =(cθ3∗L6∗cθ4)−(d4∗sθ3)+[(L5∗cθ3)+L3+d3+(P2∗tan(φ1))]

**B.**  **Inverse Kinematics (Graphical Method)**

**Figure 5:**
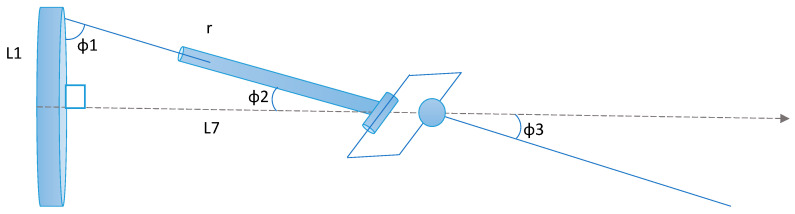
Top view of the kinematic structure.

the inverse kinematics of graphical method/approach is carried out by considering top view of the proposed kinematic structure as in Figure 5. The equations of graphical approach are as follows:

θ1 = θ2 as both is related to each other

Assume r=L2+d2+L3

From the top view, by using a right-angle triangle we can write

$${\left(r\right)}^{2}={\left(L1\right)}^{2}+{\left(L7\right)}^{2}$$

$$\begin{array}{cc} L7 & =\sqrt{{\left(r\right)}^{2}-{\left(L1\right)}^{2}}\\ & =\sqrt{{\left(L2+d2+L3\right)}^{2}-{\left(L1\right)}^{2}} \end{array}$$

tan φ2 = L1/L7 i.e., φ2 = tan^−1^ $\left[\frac{L1}{L7}\right]$

tan φ1 = L7/L1 i.e., φ1 = tan^−1^ $\left[\frac{L7}{L1}\right]$

because L6 is in the same plane of r, we can say φ2 = φ3

hence θ3 = 2*φ2 & θ4 = 2*φ3

**C.**  **Jacobian Analysis**

The Jacobian Analysis describes the relationship between the linear velocity and the angular velocity of the flapping link. The Jacobian matrix transforms the linear velocity of the flapping link space in with respect to its joint actuator space. Moreover, it is very much necessary for motion analysis and trajectory path follow-ups.

$$J=\left[\begin{array}{c} J_{V}\\ J_{w} \end{array}\right]$$

The obtained Jacobian matrix for the above derived mechanism/model considered from Figure 4 is as shown below. (note: “cos” and “sin” are indicated by “C” and “S” below)

$$J=\left[\begin{array}{cccc} -Y4 & -\left(Y4-Y1\right)\; & C_{12}\left(Z4-Z2\right) & S_{12}S_{3}\left(Z4-Z3\right)+S_{3}\left(Y4-Y3\right)\\ -X4 & -\left(X4-X1\right) & -S_{12}\left(Z4-Z2\right) & C_{12}C_{3}\left(Z4-Z3\right)+S_{3}\left(X4-X3\right)\\ 0 & 0 & -S_{12}\left(Y4-Y2\right)-C_{12}\left(X4-X2\right) & C_{12}C_{3}\left(Y4-Y3\right)-S_{12}S_{3}\left(X4-X3\right)\\ 0 & 0 & -S_{12} & C_{12}C_{3}\\ 0 & 0 & C_{12} & S_{12}S_{3}\\ 1 & 1 & 0 & S_{3} \end{array}\right]\left[\begin{array}{c} {\dot{\theta }}_{1}\\ {\dot{\theta }}_{2}\\ {\dot{\theta }}_{3}\\ {\dot{\theta }}_{4} \end{array}\right]$$

Note: C1=Cos θ1 and S1=Sin θ1

$C_{12}=Cos\;\left(\theta 1+\theta 2\right)$ and $S_{12}=Sin\;\left(\theta 1+\theta 2\right)$

$$S_{12*}=Sin\;\left(\theta 1-\theta 2\right)$$

$$J=\left[\begin{array}{c} \dot{X}\\ \dot{Y}\\ \dot{Z}\\ {\dot{\omega }}_{X}\\ {\dot{\omega }}_{Y}\\ {\dot{\omega }}_{Z} \end{array}\right]=\left[\begin{array}{c} \left(-Y4*{\dot{\theta }}_{1}\right)-\left[\left(Y4-Y1\right){\dot{\theta }}_{2}\right]+\left[C_{12}\left(Z4-Z2\right){\dot{\theta }}_{3}\right]+\left[\left(S_{12}S_{3}\left(Z4-Z3\right)+S_{3}\left(Y4-Y3\right)\right){\dot{\theta }}_{4}\right]\\ \left(-X4*{\dot{\theta }}_{1}\right)-\left[\left(X4-X1\right){\dot{\theta }}_{2}\right]-\left[S_{12}\left(Z4-Z2\right){\dot{\theta }}_{3}\right]+\left[\left(C_{12}C_{3}\left(Z4-Z3\right)+S_{3}\left(X4-X3\right)\right){\dot{\theta }}_{4}\right]\\ \left\{\left[-S_{12}\left(Y4-Y2\right)-C_{12}\left(X4-X2\right)\right]{\dot{\theta }}_{3}\right\}+\left\{\left[C_{12}C_{3}\left(Y4-Y3\right)-S_{12}S_{3}\left(X4-X3\right)\right]{\dot{\theta }}_{4}\right\}\\ \left(-S_{12}*{\dot{\theta }}_{3}\right)+\left(C_{12}C_{3}*{\dot{\theta }}_{4}\right)\\ \left(C_{12}*{\dot{\theta }}_{3}\right)+\left(S_{12}S_{3}*{\dot{\theta }}_{4}\right)\\ {\dot{\theta }}_{1}+{\dot{\theta }}_{2}-S_{3}{\dot{\theta }}_{4} \end{array}\right]$$

where,

$$X1=(P1*\;C_{1})$$

$$Y1=(P1*\;S_{1})$$

Z1=P2∗tan(φ1)

$$X2=P1[C_{1}-C_{12}]$$

$$Y2=P1[S_{1}-S_{12*}]$$

Z2=L3+d3+P2∗tan(φ1)

$$X3=(L5*C_{12}*S_{3})-\;(d3*S_{12})+P1(C_{1}-C_{12})$$

$$Y3=(L5*S_{12}*S_{3})-\;(d3*C_{12})+P1(S_{1}-S_{12*})$$

Z3=(L5∗)+L3+d3+P2∗tan(φ1)

$$X4=\mathrm{PX}=[C_{12}*S_{3}*L6*C_{4}]+[S_{12}*L6*S_{4}]+[d4*C_{12}*C_{3}]+\left\{\left[L5*C_{12}*S_{3}\right]-\left[d3*S_{12}\right]+P1\left[C_{1}-C_{12}\right]\right\}$$

$$Y4=\mathrm{PY}=[S_{12}*S_{3}*L6*C_{4}]+[C_{12}*L6*S_{4}]+[d4*S_{12}*S_{3}]+\left\{\left[L5*S_{12}*S_{3}\right]+\left[d3*C_{12}\right]+P1\left[S_{1}-S_{12*}\right]\right\}$$

$$Z4=\mathrm{PZ}=(C_{3}*L6*C_{4})-\left(d4*S_{3}\right)+[\left(L5*C_{3}\right)+L3+d3+\left(P2*\tan(\varphi 1))\right]$$

Additionally, the Jacobian matrix is determined utilizing the rotation matrix derivative, which is a vital resource for relating the joint space velocity to the flapping link velocity and interpreting nonlinearities within the working area. Likewise, the Jacobian matrix serves as the foundation for assessing Yoshikawa’s measure and calculating the Manipulability Ellipsoid, indicating the flapping link’s capacity to transform location and direction when given a joint configuration.

## 4. Kinematic Simulations and Results

**Figure 6:**
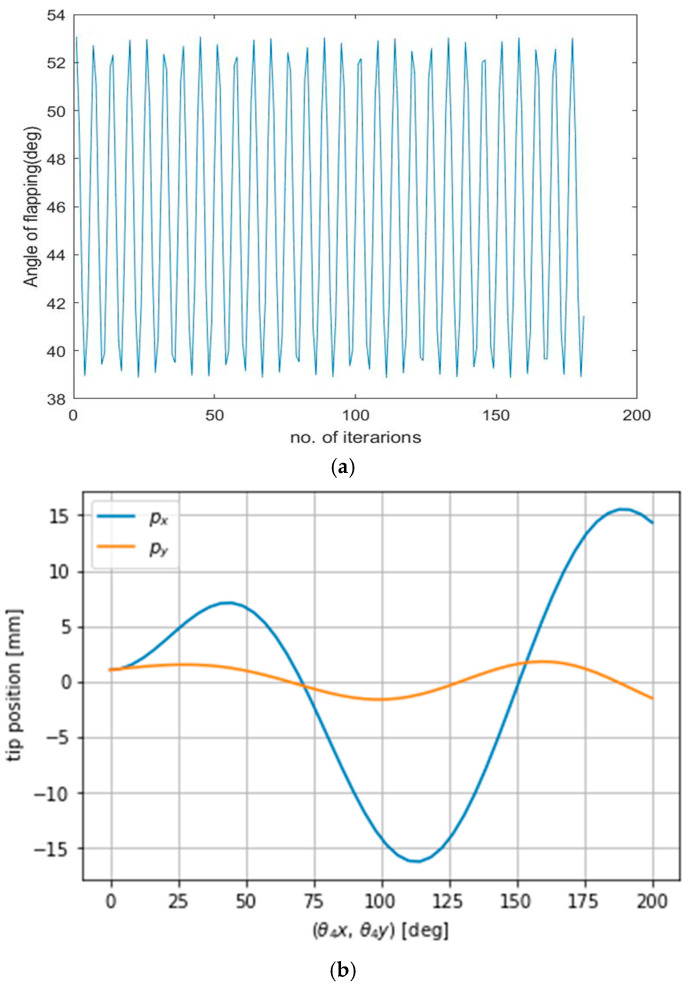

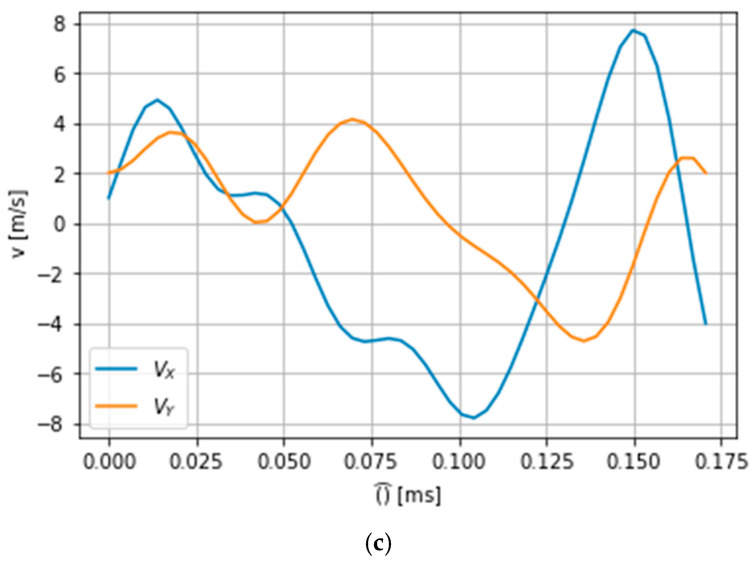
(**a**) Flapping angle obtained from the forward kinematics of the DH table. (**b**) Variation in flapping liver tip with respect to the driving crank angle in *x* and *y* direction. (**c**) Variation in liver tip velocity with respect to time.

The forward kinematic equations derived in the above section, i.e., Figure 4 and Table 1, were implemented into PYTHON programming with a controlled inputs at x, y, and z coordinates, and looping to obtain the theta 4 value as shown in Figure 6a. hence from Figure 6a, we can see that the angle of flapping obtained at theta 4 was between 39° and 53°, along with the number of iterations/samples, i.e., 53 − 39 = 14°. The PYTHON-implemented dh table was used for homogeneous transformation matrix conversions and simulations. The variation in flapping liver (22 mm length) edge/tip motion in millimeters relative to the driving crank motion in degrees is depicted in Figure 6b above. The Jacobian calculated matrix was implemented in Python as a continuation of the dh parameters, and the velocity variation of the liver tip movement with respect to time in the *x* and *y* directions was obtained. This information is depicted in Figure 6c.

**A.**  **Simscape Multibody^TM^ Model**

**Figure 7:**
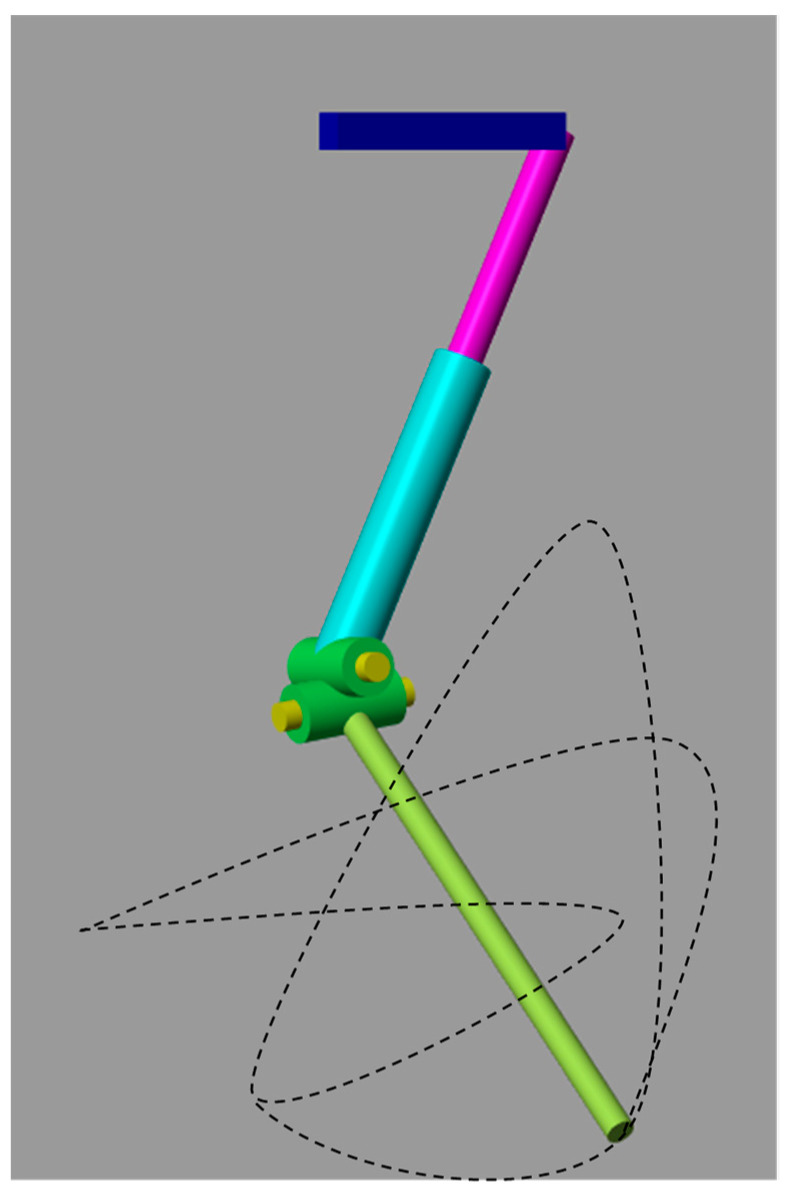
Similar model design in Simscape Multibody^TM^ MATLAB.

**Figure 8:**
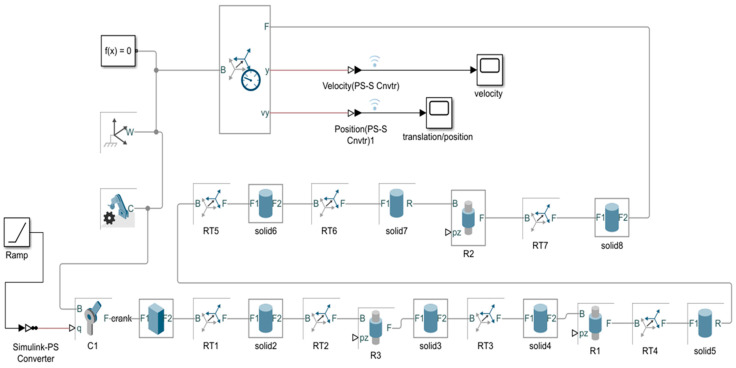
Equivalent Model for the mechanism in Simscape Multibody^TM^ (SimMechanics^TM^) of MATLAB 2021b.

For further dynamics analysis and validation of the mechanism design, the above model (Figure 4) was partially designed by leaving the rectangular frame structure in Simscape MultibodyTM as shown in Figure 7. The equivalent actuation circuit for the designed mechanism is shown in Figure 8. To demonstrate the significance of the rectangular frame structure in the proposed design, simscape multibody 3D design modelling was carried out.

Additionally, by specifying the joint coordinates, the model enabled us to calculate position, and velocity, as well as generate automatic 3D animations to visualize the system’s behavior. The individual solid blocks were selected with the aluminum material with a density of 2.7 g/cm^3^. The ramp input signal drove the first revolute joint as in the Equivalent Model shown in Figure 8.

**Figure 9:**
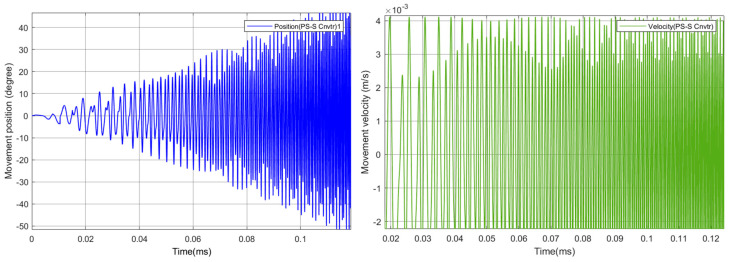
Obtained Position and Velocity of the Wing Tip.

In order to test the model and the joint’s evolution (Figure 9), the position and velocity of the flapping link was evaluated according to the direct kinematics derived in above section. To analyze the motional fluctuations of the flapping link, two cylindrical joints were connected with each other as shown in Figure 7. However, the obtained path or position of motional flapping was not symmetrical, but rather random. As a result, a rectangular framework is intended to provide a better flapping motion via the modelling of 3D mechanism’s design in Compmech GIM-2022 software. Below, Section B shows simulations which demonstrate the significance of adopting a rectangular frame structure for the desired motion direction in this design.

**B.**  **Compmech Gim Software Model**

**Figure 10:**
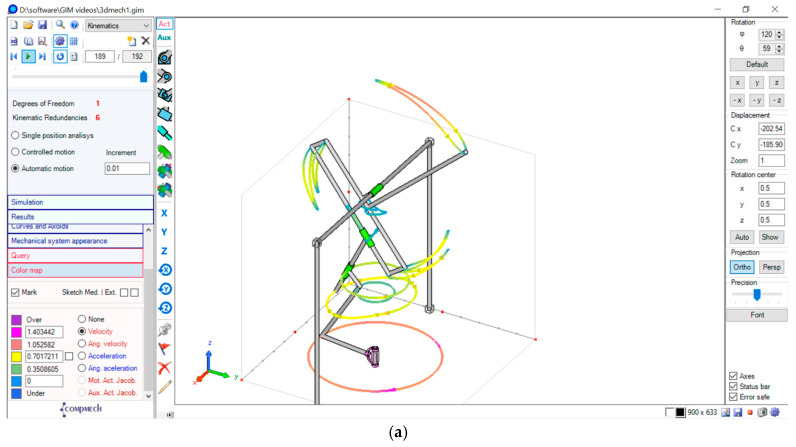

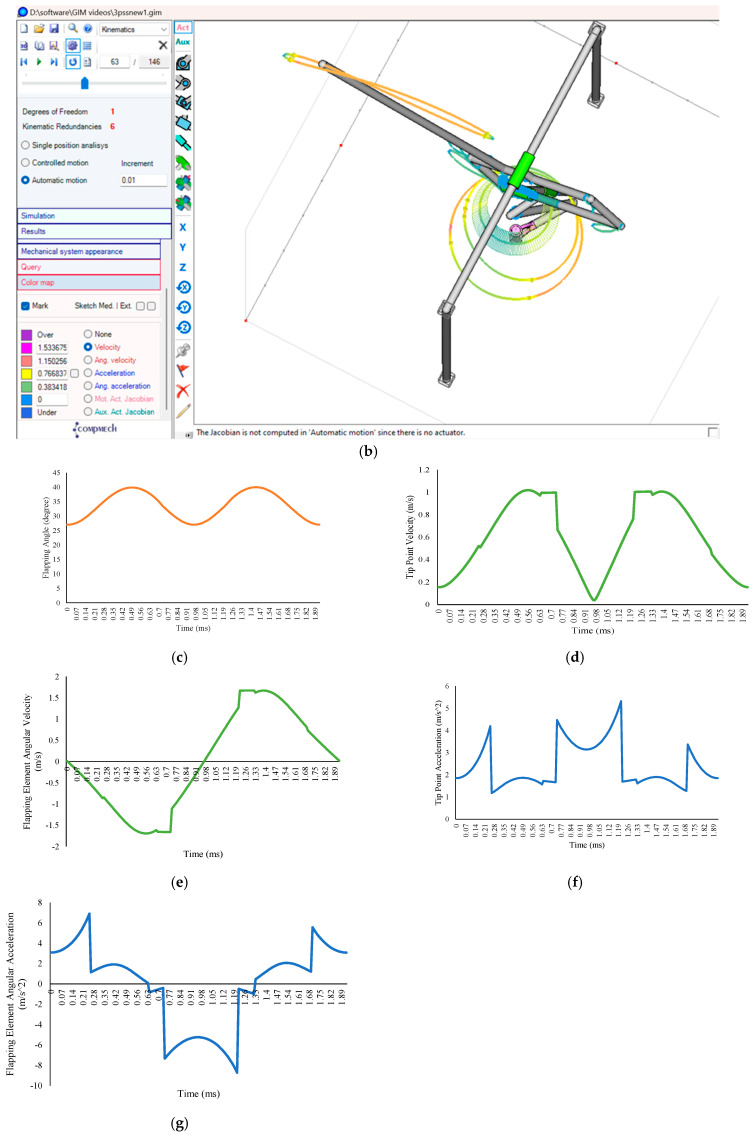
(**a**) Similar Model Design in Compmech Gim-2022 Software. (**b**) Flapping lever tip position tracing. (**c**) The angle of flapping obtained. (**d**) Flapping velocity at the flapping link. (**e**) Angular velocity of the flapping element. (**f**) Acceleration at the flapping link tip. (**g**) Angular acceleration of the flapping element.

The mechanism was also simulated using Compmech Gim Software, where kinematic redundancies considered were for 6. The workspace with the velocity across the motion path is clearly illustrated in Figure 10a through color representation from under to over as color variation with respect to change in the velocity of motion. As they descend, the flapping represents one complete wingbeat cycle. Figure 10b was created by adjusting the size of the rectangular frame and links 1, 2, and 3. It is clearly apparent that the variance in link lengths caused the final position fluctuation of the flapping lever tip. As in Figure 10a, the obtained tracing of the liver tip had a leaf-like shape, whereas the liver tip tracing in Figure 10b had a figure-of-eight shape. As shown in Figure 10c, the flapping angle obtained was the path taken by the flapping element.

The maximum angle of flapping obtained through angle between two vectors (i.e., between the alignment of final flapping element and the fixed supporting structure of Figure 10a was obtained as the flapping angle range 25–40°, as shown in Figure 10c, i.e., 40 − 25 = 15°, which is the angle of flapping obtained per wing beat cycle. The variation in wing beat angle velocity and acceleration, obtained in simulations per wing beat cycle was as shown in Figure 10, e.g., the point velocity and acceleration analyzed at the flapping link tip per wing beat cycle is as shown Figure 10d and Figure 10f, respectively. The variation in angular velocity and angular acceleration with respect to the whole lever element is depicted in Figure 10e and Figure 10g respectively.

Because the levers’ design lengths were variable, the obtained lever tip velocities of both python-programmed and comp mech-3D modelled design simulations were very similar to one another. The disparity in velocity can be reduced to an acceptable level by further refining the proposed design of connecting link sizes.

## 5. Experimental Setup and Results

**Figure 11:**
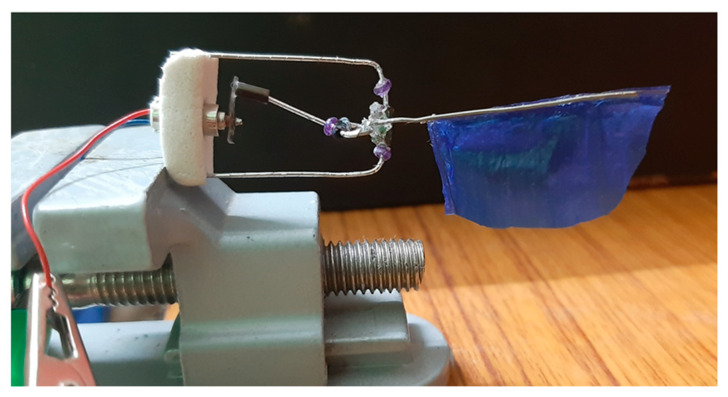
The flapping actuation mechanism’s test-bench design.

**Figure 12:**
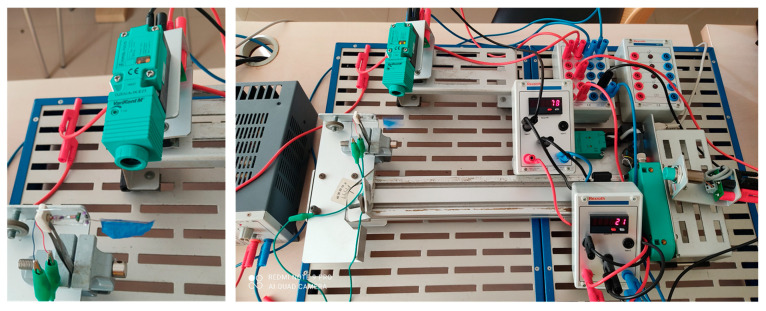
Flapping Frequency Measuring Setup.

Following simulation analysis, we were able to perform with the flapping actuation mechanism fabricated by hand as a test bench prototype, as illustrated in Figure 11. The wing was directly attached to the output link of a rotary to flapping conversion mechanism. The design possesses the minimal friction and fast mechanical transmission characteristics necessary to achieve better flapping motion. However, we could obtain max 18–20° of flapping angle through this actuation mechanism, which is similar to the simulation results. The actuator’s flapping consistency was measured through a photoelectric sensor with a counter utilized to measure its frequency as shown in Figure 12.

**Figure 13:**
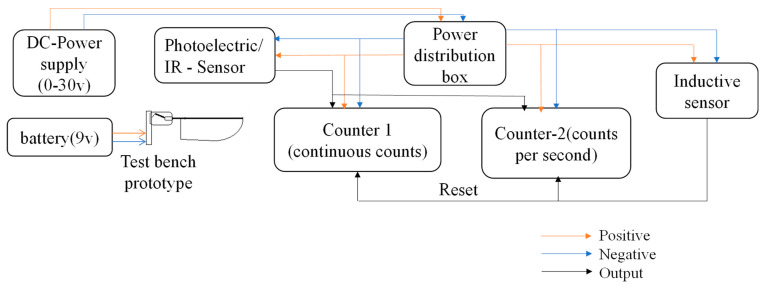
Equivalent Circuit Diagram of Flapping Frequency Measuring Setup.

We gave a dc input power of 30 v at the distribution box, and the same supply was distributed to proximity sensors and counters, as shown in Figure 12 and Figure 13. A constant 9 v battery source was applied separately to power the test bench model. Moreover, the calibration of sensors was taken care of during the initial tests. When the infrared sensor detects wing motion, it begins transmitting its output to counters. Counter-1 counts the detected flaps continuously by displaying them, and counter-2 is programmed to count the number of flaps/counts per second, i.e., frequency. Additionally, the inductive sensor’s output resets the values of the counters.

**Figure 14:**
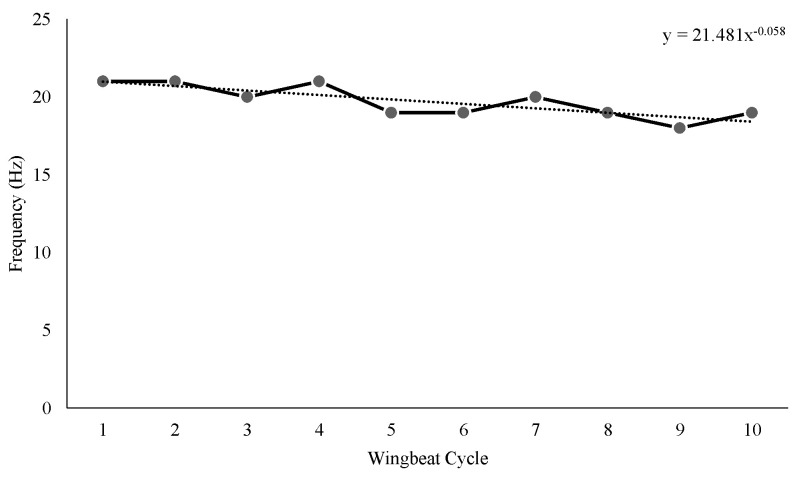
Measured Flapping Frequency.

**Figure 15:**
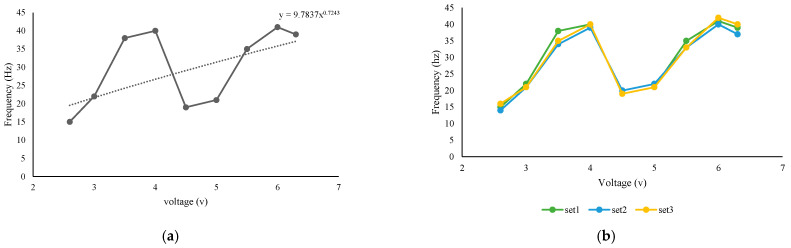
(**a**). Variation in flapping frequency as a function of increased input voltage. (**b**). Multiple set of tests for flapping frequency analysis.

According to Figure 14, we obtained a flapping frequency variation of between 18 and 21 hz for a 9v input voltage. The wing motion was clearly distinct relative to the stroke line, i.e., the wing beat frequency decreases concurrently (Figure 14). The frequency plot clearly shows a significant decrease/variation in frequency with respect to wing-beat cycles. This was strongly associated with the variation in flapping wing velocity as well. However, when we varied the input voltage, the corresponding flapping frequency fluctuated. As illustrated in Figure 15a, as the input voltage increased from 2.6 v to 4 v, the flapping frequency also increased and reached 40 hz. However, when the input voltage was 4.5 volts, the flapping frequency dropped abruptly to 19 hz and then rose to 40 hz as the input voltage increased to 6 v.

Due to the fact that the obtained flapping frequency was not linear with increasing voltage, we repeated the real-time experiments to obtain multiple sets of readings for the same. As shown in Figure 15b, we obtained a similar wave graph of flapping frequency variation as a function of the increasing voltage. As a result of Figure 14 and Figure 15, we can conclude that the design mechanism produced a higher flapping frequency by lowering the input voltage. However, the abrupt decrease in flapping frequency at 4.5 v remains a puzzle, which we are still attempting to solve. In general, however, the required battery voltage for MAVs was 3.5 volts when considering their mass limits. Accordingly, the obtained flapping frequency up to 3.5 volts is more than adequate. Hence the proposed mechanism is suitable for flapping wing drones’ application.

**Figure 16:**
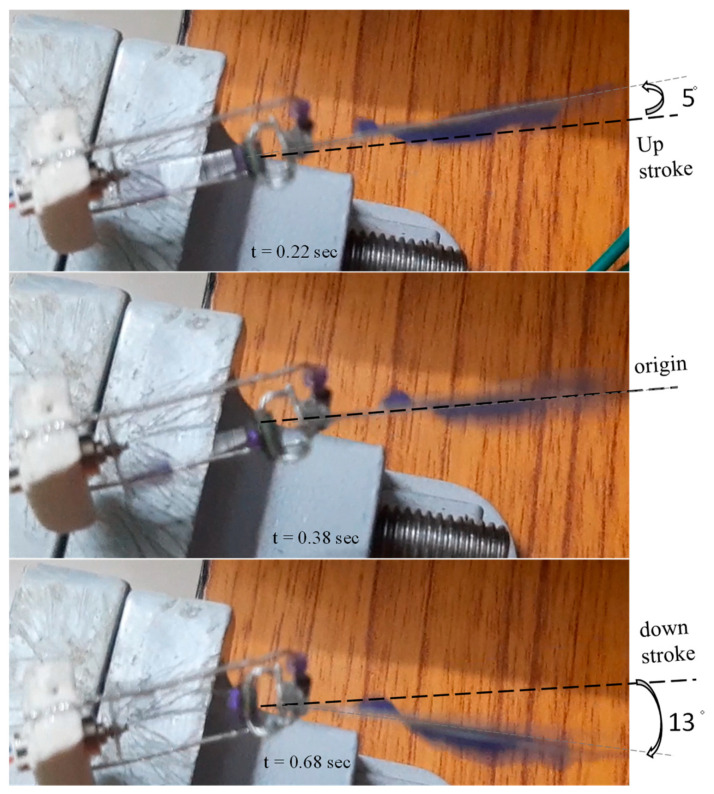
Sequential flapping captured frames of the proposed mechanism.

The successive frames of the proposed mechanism for low flapping frequency are also depicted in Figure 16. When 3.5 volts was supplied to capture the flapping action, frames were collected every 0.20 s. At separate times, we were capable of observing the changing angular movements of both wings. As an extension of this research work for the future, it is intended to publish the additional experimental analysis with a parametric study for thrust and lift generation with variable set, size, and material of wings. This will be completed as part of the parametric study.

**Figure 17:**
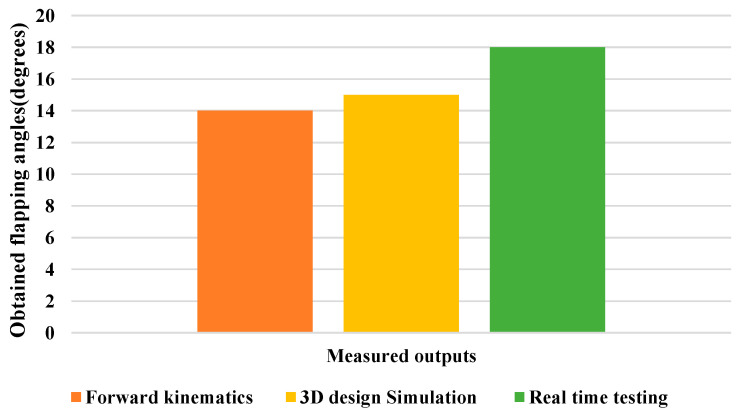
Comparison of obtained flapping angle from forward kinematics to real time testing.

The maximum displacement of the flapping wing was determined by using a distance measurement sensor, which was then transformed into an angle before being checked against the structural flappable workspace in both the vertical and horizontal directions. As can be seen in Figure 16, this was achieved by flapping the wing at an angle ranging from 18° to 20°. It was the angle of flapping that was obtained for each cycle of the wing beat. The proposed design mechanism’s structural specifications are as outlined in Table 2, which may be seen here. Additionally, a comparison of the observed outputs of all of the forward kinematics, 3D design simulation, and real time testing was carried out for the flapping angle as shown in Figure 17. It can be seen that there were not many variances in outputs, and there was similarity in all of the angles obtained through flapping. Because the overall prototype design was made with maximum possible reduction in its size, the obtained flapping angle was 18°; if the connecting links sizes were varied, the angle of flapping could be varied. This effort has produced a simple model that can aid in the design of FWMAVs inspired by insects. FWMAVs at centimeter or millimeter length scales are typically propelled by piezoelectric, dielectric/electrostatic elastomers, electromagnetic, or other reciprocating actuators. In any event, the rigidity of the actuator itself resembles that of the insect thorax most closely. Slight displacements of links in actuator can be converted into wing rotation by a mechanical transmission that acts in a manner similar to the insect wing hinge. Hence the proposed mechanism is resizable depending on the requirements.

## 6. Conclusions

The current article discussed efforts to construct a revolutionary test bench model of flapping wing actuation mechanism with the goal of applying it to bionic MAVs. We presented the kinematic analysis of the Crank driven-triple cylindrical slidable and rotatable joint mechanism arrangement with direct and inverse kinematics and also Jacobian representation for linear velocity and angular velocity measurements in this article. This mechanism can be qualified to a specific family of the spatial one-DOF linkage mechanisms which contain triple cylindrical joints perpendicular to one another in a horizontal plane. We performed 3D simulations of the mechanism design modelling to determine the flapping motion performance measurement and track the position/path of flapping link tip with two different types of software. A novel flapping wing actuation mechanism on a Crank driven-triple cylindrical slidable and rotatable joint technique was designed, and a simulation of motion analysis was performed. The simulated results followed the mechanism’s expected kinematics. The developed kinematic model can determine the mechanism’s design parameters and delivers the benefit of scalability in MAV design. Effective modelling and simulation have established a strong foundation for future research into dynamic analysis and mechanism manufacture. After solving the dynamic equations, we will implement control techniques in the next stage.

## Figures and Tables

**Table 1:** Denavit–Hartenberg (DH) table.

n	$d_{z\left(i-1\right)}$	$\theta_{z\left(i-1\right)}$	$\alpha_{x\left(i\right)}$	$r_{x\left(i\right)}$
1	P2∗tan(φ1)	θ1	0	P1
2	L3 + d2	θ2	−90˚	−P1
3	±d3	−90˚ + θ3	−90˚	L5
4	±d4	θ4	0	L6

**Table 2:** Design specifications of the proposed test bench prototype.

**Over all Mass**	1.6 g (Mechanism + Wing + Motor + Supporting Structure)
**Motor weight**	0.60 g
**Wing span**	36 mm
**Wing area**	3.6 × 2 (L × B)
**Flapping frequency**	38 hz
**Input voltage**	3.5 v DC
**Angular velocity**	ω1 = 0.401426/0.46 = 0.87 (rad/s)

## Nomenclature

*n*	Link index
J	Joint coordinate
I	3 × 3 Link inertial matrix
$T_{n}^{n-1}$	4 × 4 Transformation matrix of assumed coordinate nodes/systems from link *n* − 1 to *n*
$T^{\left(Jn\right)}\;$	4 × 4 Transformation matrix of standard reference coordinate system
$R^{\left(Jn\right)}$	3 × 3 Rotation matrix of assumed coordinate nodes/systems from link *n* to *n* − 1
$O^{\left(Jn\right)}$	Origin of the of assumed coordinate systems from link *n* to *n* − 1
$\psi^{\left(Jn\right)}$	Joint coordinate vector from link *n* to *n* − 1
$q^{\left(Jn\right)}$	Generalized coordinates from link *n* to *n* − 1
Ln & Pn	Link length
dn	the translation movement at the joint
$d_{z\left(i-1\right)}\;$	Translation along *z*-axis when *i* = 1, 2, 3… depending on the frame (i) consideration
$\theta_{z\left(i-1\right)}$	Rotational angle about *z*-axis when *i* = 1, 2, 3… depending on the joint (i) consideration
$\alpha_{x\left(i\right)}$	Rotational angle about *x*-axis when *i* = 1, 2, 3… depending on the joint (i) consideration
$r_{x\left(i\right)}\;$	Translation along *x*-axis when *i* = 1, 2, 3… depending on the frame (i) consideration
